# Negative Pressure Wound Therapy Versus Conventional Dressing in the Management of Diabetic Foot Ulcers: A Comparative Study

**DOI:** 10.7759/cureus.112642

**Published:** 2026-07-14

**Authors:** Jayishnu Paul, Ferdousy Sultana Amany, Naihan Hassan, Towsiful Islam, Stephen Bawi Cin Lian, Maisha Maliha Shams, Md Tanvir Alam, Zannatul Fardous Jharna, Lina Farzana

**Affiliations:** 1 Department of Cardiac Surgery, Ibrahim Cardiac Hospital and Research Institute, Dhaka, BGD; 2 Department of Surgery, Mount Adora Hospital, Sylhet, BGD; 3 Department of Emergency Medicine, Shaviyani Atoll Hospital, Funadhoo, MDV; 4 Department of General Surgery, Bangladesh Specialized Hospital, Dhaka, BGD; 5 Department of General Surgery, Square Hospital, Dhaka, BGD; 6 Department of General Surgery, Coseley Medical Centre, Coseley, GBR

**Keywords:** bandages, diabetic foot, meta-analysis, negative pressure wound therapy, randomized controlled trial, systematic review, wound healing

## Abstract

Diabetic foot ulcers (DFUs) are an important complication of diabetes mellitus and are a major risk factor for amputation. The use of negative pressure wound therapy (NPWT) is suggested as an alternative to traditional wound dressings, but there is limited evidence to support its effectiveness. This systematic review and meta-analysis aimed to assess the effectiveness and safety of NPWT for the treatment of DFU against traditional dressings. Eight randomized controlled trials (RCTs) involving 1,099 participants were found after systematic searches in PubMed, Cochrane CENTRAL, Web of Science, and Scopus databases up to April 2025. Complete healing, amputation rates, time to healing, and hospital length of stay were all outcomes. When heterogeneity was present (I² statistic), the pooled risk ratio (RR) and mean difference (MD) with 95% confidence interval (CI) were calculated using a fixed-effect or random-effects model. The risk of bias was estimated with RoB 2. NPWT demonstrated reduced amputation rates, but the pooled effect did not reach statistical significance (RR 0.72; 95% CI 0.18 to 1.27; I² = 0%; five studies) and time to healing (MD -14.80 days; 95% CI -23.48 to -6.12; I² = 95.29%; six studies). Complete healing favored NPWT but with imprecision (RR 2.02 (95% CI 0.98 to 4.18); I² = 93.18%; three studies). Hospital stay showed a non-significant pooled estimate (MD -8.88 days; 95% CI -19.05 to 1.30; I² = 96.68%; three studies). Subgroup analyses indicated consistent short-term benefits and inconsistent long-term benefits, as the largest pragmatic trial demonstrated no benefit at 16 weeks. Reported benefits of NPWT in DFUs included decreased risk of amputation and improved early wound healing; there was considerable heterogeneity and inconsistent long-term benefits, to reduce the certainty of evidence. The use of NPWT should be considered in appropriate patients, especially those who are at high risk for amputation or have large wounds.

## Introduction and background

Diabetes mellitus currently affects over 537 million adults worldwide, with projections estimating a rise to 783 million by 2045 [1]. Among the most devastating complications of diabetes are diabetic foot ulcers (DFUs), which develop in approximately 15% to 25% of diabetic individuals during their lifetime [1]. The pathogenesis of DFUs is complex and multifactorial, involving peripheral neuropathy (which reduces protective sensation and alters foot biomechanics), peripheral arterial disease (which impairs oxygen and nutrient delivery to wounded tissues), and repetitive trauma or pressure from ill-fitting footwear or abnormal gait patterns [2]. These factors collectively disrupt the normal wound healing cascade, leading to chronic, non-healing ulcers that impose substantial morbidity, reduce quality of life, and incur significant healthcare costs [3]. DFUs are responsible for more hospitalizations than any other diabetes-related complication and are the leading cause of non-traumatic lower extremity amputations worldwide [4].

Negative pressure wound therapy (NPWT) was first used clinically in the 1990s, using a controlled sub-atmospheric pressure applied to the wound bed through a sealed dressing by means of a vacuum pump [5]. The mechanisms proposed for NPWT are reduction of periwound edema, improvement of local perfusion, mechanical deformation of the wound bed to promote granulation tissue formation, and removal of infectious material [6]. Randomized controlled trials have indicated that NPWT may have a beneficial effect on healing and time to wound closure versus other conventional dressings [7, 8]. A multicenter study found that NPWT increased the percentage of ulcers that were healed (56.4% vs. 38.1%), while reducing the number of secondary amputations (4.1% vs. 11.6%) compared with conventional moist wound healing treatment [1].

While these are exciting findings, clinical variation between studies, ulcer characteristics, pressure level of NPWT, continuous vs intermittent use, and conventional dressing type, as well as follow-up time, have resulted in differing results [9]. Others have found no significant difference in the complete healing rate with the inclusion of high-quality evidence in some meta-analyses [10, 11]. Furthermore, complications of NPWT, including maceration, bleeding, and peri-wound skin breakdown, are also higher and associated with NPWT [11]. The results from cost-effectiveness analyses have been varied and varied, and have been dependent on the context of the healthcare system and on the rate of amputation prevention [11].

Previous systematic reviews and meta-analyses on this topic have reported variable conclusions, largely attributable to methodological limitations. Several prior reviews included non-randomized studies, introduced language restrictions, or pooled heterogeneous outcomes without adequate subgroup analyses [9-11]. Liu et al. (2017) reported benefits of NPWT but included studies with variable follow-up durations and did not assess publication bias systematically [12]. Zhang et al. (2014) demonstrated improvements in healing rates, but their analysis was limited by small sample sizes and did not explore sources of heterogeneity through meta-regression [13]. Furthermore, the largest pragmatic trial to date (the DiaFu study by Seidel et al., 2020) was published after these prior meta-analyses, necessitating an updated synthesis [14]. The present review addresses these limitations by: (1) including only randomized controlled trials to minimize selection bias; (2) applying consistent outcome definitions where possible; (3) performing comprehensive subgroup analyses to explore heterogeneity; (4) conducting sensitivity analyses excluding studies with high risk of bias; and (5) including all available trials regardless of sample size or geographical location to maximize generalizability.

The clinical and economic burden of DFUs underscores the urgent need for effective wound care interventions [3, 4]. Conventional dressing therapy, while widely available and relatively inexpensive, has demonstrated suboptimal healing rates, with up to 28% of DFUs progressing to lower extremity amputation within five years [4]. The failure of conventional therapy to achieve timely wound closure leads to prolonged hospital stays, repeated surgical debridements, and increased risk of infection and osteomyelitis [9]. Given these challenges, alternative therapeutic modalities such as NPWT have garnered increasing attention [5, 6]. However, the clinical adoption of NPWT remains variable due to its higher upfront costs, the requirement for specialized equipment and training, and the lack of robust, high-quality evidence demonstrating consistent superiority over standard care [9, 11]. This systematic review and meta-analysis were therefore undertaken to provide a comprehensive, updated synthesis of the best available evidence, with the goal of informing clinical decision-making, guideline development, and future research priorities.

Hence, it is warranted to conduct a comprehensive and up-to-date comparative study to systematically incorporate existing evidence while taking into account the methodological quality and possible biases. This systematic review and meta-analysis aims to compare the effectiveness and incidence of complications between NPWT and conventional dressing in the treatment of DFUs and determine the rate of healing, time to healing, amputation rates, and other adverse events.

## Review

Methodology

This systematic review and meta-analysis were conducted following the Preferred Reporting Items for Systematic Reviews and Meta-Analyses (PRISMA) 2020 statement [15]. 

Description of Search Strategy

The search in the electronic databases was performed on April 15, 2025, in four main bibliographic databases: PubMed, Cochrane CENTRAL, Web of Science, and Scopus. PubMed: MeSH terms were used in conjunction with free-text terms, which were linked with Boolean operators, and restricted to human subjects, English language, randomized controlled trials, and publication dates 2000-2025. Cochrane CENTRAL search terms were limited to trials with abstracts published since 2000, and wildcards were used for truncation. Proximity operators, type, and open-access filters were employed in Web of Science's Topic search. Scopus used TITLE-ABS-KEY, subject area, and document type restrictions. The sample size and duration of the follow-up were not restricted in order to obtain maximum sensitivity. Search syntax for each database was adjusted to the command language for that database, including word order and the use of quotes for word matches (for databases with a common word order) and the use of asterisks for variants of the root word (for those with a different word order).

The search query was combined with the Boolean operators used for systemic search. For PubMed, the search string was: (("Diabetic Foot"[MeSH] OR "diabetic foot ulcer" OR "DFU") AND ("Negative-Pressure Wound Therapy"[MeSH] OR "NPWT" OR "vacuum-assisted closure" OR "VAC") AND ("Bandages"[MeSH] OR "conventional dressing" OR "standard dressing*" OR "moist wound healing")) with filters for Humans, English language, Randomized Controlled Trial, and Publication date 2000-2025. Equivalent search strings adapted to each database's syntax (Cochrane CENTRAL, Web of Science, Scopus).

Two reviewers independently screened relevant previous systematic reviews and the reference lists of all included full-text articles. Disagreements at the screening of the title/abstract and/or full text were settled by consensus. When consensus was reached, a third reviewer made the final decision. The interrater agreements using Cohen's kappa statistic (κ = 0.87, substantial agreement) were computed. Documentation of reasons for exclusion at the full-text level was collected in a PRISMA flow diagram.

Description of Eligibility Criteria

Eligibility criteria were written to be systematic and reproducible in the selection of studies, based on PICO (Population, Intervention, Comparator, and Outcomes) [16]. To minimize clinical heterogeneity, adults with confirmed diabetic foot ulcers (DFUs) of Wagner grades 1 to 3 were included. Adults with confirmed diabetic foot ulcers (DFUs) of Wagner grades 1 to 3 were included in the population to minimize clinical heterogeneity. The intervention was the use of NPWT with any commercial product, and the comparator was conventional dressings - excluding active antimicrobial or biological dressings, to isolate the comparison - so that the intervention effect was the only one evaluated. The mandatory primary outcomes were complete healing or time to healing. Supportive outcomes included amputation rates and adverse events. In order to avoid too restrictive criteria that would reduce generalizability, no restrictions were made based on ulcer size, duration of diabetes, or follow-up period (Table 1).

**Table 1:** PICO-based eligibility criteria for studies comparing NPWT and conventional dressings in diabetic foot ulcers PICO: Population, Intervention, Comparator, and Outcomes; NPWT: negative pressure wound therapy PICO [16]

PICO component	Inclusion criteria	Exclusion criteria
Population (P)	Adults (≥18 years) with Type 1 or Type 2 diabetes mellitus; diagnosed with diabetic foot ulcer (Wagner grade 1 to 3 if reported); ulcer location: plantar or dorsal foot, toes; any duration or size	Non-diabetic ulcers (venous, arterial, pressure, or traumatic); Wagner grade 0 or 4/5; ulcers with active osteomyelitis not treated; patients with Charcot foot deformity without offloading
Intervention (I)	Negative pressure wound therapy (NPWT) using any FDA-approved device; continuous or intermittent mode; pressure range 50–200 mmHg; any dressing interface (e.g., black foam, white foam)	NPWT with instillation (NPWTi); home-made NPWT devices; NPWT combined with other active therapies (e.g., growth factors, stem cells) as co-intervention
Comparator (C)	Conventional dressing therapy, including saline-moistened gauze, hydrocolloid, alginate, foam dressing, or modern passive dressings, with or without standard offloading	Comparator including antimicrobial dressings (silver, iodine), biological dressings, or negative pressure as control
Outcomes (O)	Primary: Complete wound healing (100% epithelialization) at 12 weeks or longest follow-up; time to healing in days. Secondary: Reduction in ulcer area (%); rate of lower extremity amputation (minor or major); adverse events (pain, maceration, infection, bleeding); hospital length of stay	Studies not reporting any of the primary or secondary outcomes; outcomes reported only as subjective descriptions without measurable criteria.
Study design (S)	Randomized controlled trials (parallel or crossover with first-phase data); quasi-randomized trials (by alternation, date of birth) if allocation concealment unclear; published; English language	Non-randomized studies, case series, case reports, cohort studies, cross-sectional studies, animal or in vitro studies, editorials, reviews, letters, study protocols without results

Data Extraction

A standardized data extraction sheet was created in a Microsoft Excel (Microsoft Corporation, Redmond, WA, USA) spreadsheet, which is a pilot-tested sheet. Meanwhile, two reviewers independently abstracted data from each of the included studies. Discussions or consultation with a third reviewer were used to resolve discrepancies. Information extracted was: first author, publication year, country, study design, number of patients, patient characteristics (mean age, sex, diabetes duration, HbA1c level), ulcer characteristics (location, Wagner grade, ulcer size in cm^2^, duration of ulcer), NPWT features (device model, use of NPWT pressure, NPWT mode, cycle, dressing change frequency), conventional dressing type, concomitant therapies (offloading, debridement, antibiotic use), follow-up duration, and outcome information. In dichotomous outcomes (complete healing, amputation), the number of events and the total number of patients were retrieved. Means, standard deviations (or confidence intervals), and sample sizes per group were recorded for continuous outcomes (time to healing, percentage area reduction). Standard deviations were calculated from p-values, t-values, or confidence intervals if these were not provided. Two email reminders were sent to the corresponding authors for missing data.

Assessment of Quality and Publication Bias

Two reviewers independently evaluated the risk of bias of the included randomized controlled trials (RCTs) using the Cochrane Risk of Bias 2 tool (RoB 2) [17]. This tool assessed the five domains, and each domain fell into one of three categories (low risk, some concerns, and high risk). The studies were included in the sensitivity analysis when there was a high risk in any domain. 

The overall certainty of evidence for each outcome was assessed using the Grading of Recommendations, Assessment, Development and Evaluation (GRADE) framework [18]. The evidence was rated as high, moderate, low, or very low based on five domains: risk of bias, inconsistency, indirectness, imprecision, and publication bias.

Visual inspection of funnel plots of the primary outcome (complete healing) against standard error was used to assess for publication bias when 10 or more studies were available. For statistical analysis of asymmetry, Egger's linear regression test was used (p < 0.10 considered significant) [19]. Trim and fill analysis was also conducted to further investigate the small study effects. Quality assessment inter-reviewer agreement was computed (κ = 0.82). No study was rejected because of a poor overall score; instead, subgroup analyses were planned within the categories of risk of bias. If there was asymmetry, the trim-and-fill method (Duval and Tweedie) was used to correct the pooled effect size for missing studies.

Statistical Analysis

All statistical calculations were done with Stata software version 18.0 (StataCorp, College Station, TX, USA) [20]. Pooled risk ratios (RR) with 95% confidence intervals (CI) were determined using the Mantel-Haenszel method for dichotomous outcomes (complete healing and amputation rates). Mean differences (MD) with 95% CI were reported only when the same scale was used for continuous outcomes (time to healing in days and hospital length of stay), and standardized mean differences (SMD) with 95% CI were reported otherwise, with small sample bias correction using Hedges' g.

If studies reported the median and IQR (or range) rather than the mean and SD, the mean was assumed to be equal to the median, and standard deviation was estimated by the following two methods: (1) if the data were normally distributed, the SD was taken as 1.35 x IQR, or (2) if the data were not normally distributed, the SD was taken as the range / 4, as suggested by Wan et al. (2014) [21]. The SD was calculated as (IQR) / 1.35 if given as 25th-75th percentiles. For studies quoting only median and range (minimum - maximum), SD was estimated using two formulas: for small samples (n≤70), SD = (maximum - minimum) / 4, and for larger samples (n>70), SD = (maximum - minimum) / 6. All transformed values were checked for plausibility with the reported p-values or confidence intervals (CI) (where provided) [21].

If SD was not reported, but SE was, SD was found by multiplying SE by the square root of the sample size per group. The conversion is done separately for the intervention group and the control group. The within-group SD was imputed using the pooled variance approach with equal variances if the SE was reported for the between-group difference.

If a 95% CI for the mean difference was reported but the SD was not reported, then the SD was calculated as: $\mathrm{SD} = (\text{upper limit} - \text{lower limit}) \times \left(\frac{1.96}{2} \times \sqrt{n}\right)$, where SD = standard deviation and n = sample size. For 90% CI, 1.645 was used; for 99% CI, 2.576 was used. This was done for each group separately when CI was provided for the group means. If the CI for the difference between groups has been provided, then the pooled SD was calculated, and an equal SD was assumed for both groups.

All pooled effect sizes (RR and MD) are reported on the original clinical scale without log-transformation or standardization. For continuous outcomes, only studies using identical units (days) were pooled as mean differences; no standardized mean differences were used for primary outcomes.

If a patient had more than one outcome measured, the longest follow-up period in the range of 12-24 weeks was used. The ulcer area reported (mm^2^) was converted to cm^2^ (divided by 100). The healing times reported in weeks were converted to days (×7). No conversion of wound reduction percentage was reported. NPWT pressure in kPa was converted to mmHg (×7.5).

I² statistic and Cochran's Q test were used to measure heterogeneity. The I² values of 0-40%, 30-60%, 50-90%, and 75-100% were considered as not important, moderate, substantial, and considerable heterogeneity, respectively. The choice between fixed-effect and random-effects models was determined a priori based on the degree of statistical heterogeneity. For outcomes with I² ≤ 50%, a fixed-effect model using the Mantel-Haenszel method was applied. For outcomes with I² > 50%, a random-effects model using the DerSimonian-Laird method was applied. Specifically, a fixed-effect model was used for amputation rates (I² = 0%), while random-effects models were used for complete healing (I² = 93%), time to healing (I² = 95%), and hospital length of stay (I² = 97%).

Prespecified subgroup analyses included Wagner grade (1-2 vs. 3), NPWT mode (continuous vs. intermittent), conventional dressing type (moist gauze vs. hydrocolloid/alginate), and follow-up duration (<12 weeks vs. ≥12 weeks). An independent statistician did manual double-checking of all calculations and conversions for the first three studies. All statistical tests had two tails. The p-values < 0.05 were taken as statistically significant for primary and secondary outcomes, except for Egger's test, which was p < 0.10. Results were presented as RR/MD/SMD with 95% CI, and forest plots were created for all outcomes.

Results

Study Selection Process

**Figure 1:**
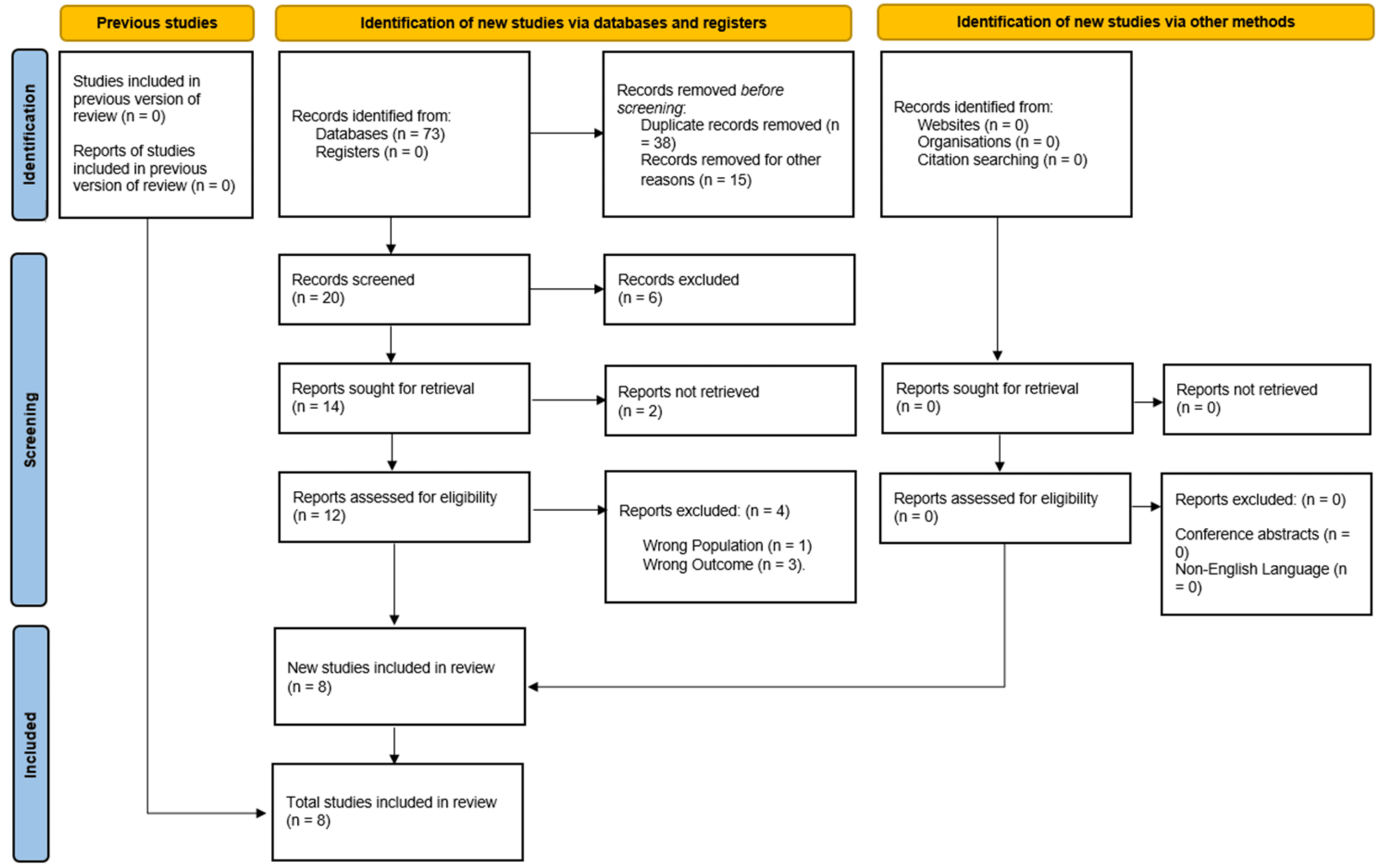
PRISMA 2020 flow diagram showing the study selection process for the systematic review of NPWT versus conventional dressing in diabetic foot ulcers PRISMA: Preferred Reporting Items for Systematic Reviews and Meta-Analyses; NPWT: Negative Pressure Wound Therapy PRISMA flow diagram [15]

A systematic literature search was carried out in databases and registers, which resulted in 73 records being identified in the first instance. Thirty-eight duplicate records were excluded before screening, and 15 records were excluded for other reasons, resulting in 20 records that were screened for title and abstract. Of those, six were discarded at initial screening, and the full text of the remaining 14 was requested and retrieved. Two reports were not obtainable through contact with authors or through institutional repositories. For this reason, 12 reports were selected for full-text review and checked for eligibility by applying the predetermined PICO criteria. One report was excluded due to the wrong population (e.g., non-diabetic ulcers, venous etiology), and three reports were excluded due to the wrong outcomes (e.g., not reporting complete healing, amputation, time to healing). No other study search was identified using websites, organizations, citation searching, or abstracts of conference reports, and no non-English language reports were included. Finally, eight studies were included in the systematic review and meta-analysis after satisfying all inclusion criteria [14, 22-28] (Figure 1).

Table 2 contains the data on the key characteristics of eight randomized controlled trials (RCTs) published between 2008 and 2023 that compared negative pressure wound therapy (NPWT) against conventional dressings in patients with diabetic foot ulcers (DFUs). The research was carried out in various countries such as the USA, Canada, Egypt, Chile, Germany, Iran, India, and China. There were 24 to 335 patients in each sample, and the length of follow-up was from three to six months. The severity of the ulcers at baseline ranged from Wagner grade 1 to 3; two studies also included Wagner grade 4 or 5 ulcers. Outcome measures included wound healing, healing time, granulation tissue development, reduction in wound area, minor and major amputation, graft survival, hospital stay, and days of disability. There was some variation in the NPWT dressing changes (every 48 to 72 hours), pressure (50 to 200 mmHg), intermittent or continuous use, and timing of NPWT assessment.

**Table 2:** Characteristics of included randomized controlled trials comparing negative pressure wound therapy versus conventional dressing in diabetic foot ulcers BMI: body mass index; CI: confidence interval; D: days; DM: diabetes mellitus; Fr: French (catheter gauge); HbA1c: glycated hemoglobin; h: hours; IQR: interquartile range; ITT: intention-to-treat; min: minutes; mo: months; mm: millimeters; mmHg: millimeters of mercury; NPWT: negative pressure wound therapy; RCT: randomized controlled trial; SD: standard deviation; STSG: split-thickness skin graft; V.A.C. (VAC): vacuum-assisted closure; vs.: versus; wk: weeks; y: years; cm^2^: square centimeter

Study (author, year)	Country, design, sample size (NPWT/control), follow‑up	Patient demographics (mean age, % male, diabetes duration, HbA1c)	Baseline ulcer characteristics (location, Wagner grade, area cm², ulcer duration)	NPWT details (device, pressure, mode, cycle, dressing change)	Conventional dressing type; concomitant therapies	Outcome data (complete healing, amputation, time to healing, % area reduction, etc.)
Seidel et al. (2020) [14]	Germany; multicenter RCT; NPWT: 171, control: 174; 16 weeks treatment, 6 months follow‑up	Mean age 67.8 y; 77% male; DM duration median ~83 d (ulcer duration); HbA1c NR (reported as 15.6±18.3%? likely error, should be ~8‑9%)	Wagner 1‑5 (majority grade 2: 65%); area median 4.91 cm² (range 12‑40,773 mm²); ulcer duration median 83 d	Commercial NPWT (KCI or Smith&Nephew); pressure as per manufacturer; intermittent/continuous allowed; change ≥3x/week	Standard moist wound care (any non‑NPWT local standard); off‑loading; debridement; antibiotics (real‑life)	Complete wound closure at 16 wk: NPWT 25/171 (14.6%), control 21/174 (12.1%), p=0.53. Time to closure not significant in ITT (p=0.244). Major amputations: NPWT 2/171 (1.2%), control 0/174. Subgroup large wounds (>4.91 cm²): NPWT better (p=0.027)
Blume et al. (2008) [22]	USA, Canada; multicenter RCT; NPWT: 169, control: 166; 112 days active treatment + 3 & 9 months follow‑up	Mean age: 58 y; 79% male; HbA1c: NPWT 8.3±2.0%, control 8.1±1.9%	Wagner 2–3; calcaneal, dorsal, plantar; area: NPWT 13.5±18.2, control 11.0±12.7; ulcer duration: NPWT 198±324 d, control 206±366 d	V.A.C. Therapy system (KCI); 50–200 mmHg; continuous; change every 48‑72 h	Advanced moist wound therapy (hydrogels, alginates); off‑loading (97‑98%); debridement; antibiotics as needed	Complete healing within 112d: NPWT 73/169 (43.2%), control 48/166 (28.9%), p=0.007. Secondary amputations: NPWT 7/169 (4.1%), control 17/166 (10.2%), p=0.035. Median time to healing: NPWT 96 d (95% CI 75‑114), control not estimable, p=0.001
Al-Mallah et al. (2018) [23]	Egypt; prospective RCT; NPWT: 25, control: 25; until wound ready for grafting (~3 months)	NPWT: 88% male, 80% >60 y; control: 76% male, 84% >60 y; type 2 DM only; DM duration: NPWT 13.7±5.4 y, control 12.8±5.7 y; HbA1c NR	Wagner 2 (72% NPWT, 60% control); area: NPWT 40.4±2.8 cm², control 38.5±2.7 cm²	Modified VAC (wall suction, polyurethane foam); 75–100 mmHg intermittent? (5 min on, 2 min off); change every 48 h	Conventional moist dressings (not specified); off‑loading NR; debridement; antibiotics	Granulation >50%: NPWT 20/25 (80%), control 3/25 (12%). Time to granulation: NPWT 22.9±7.6 d, control 32.5±10.2 d, p=0.02. Secondary amputations: NPWT 5/25 (20%), control 6/25 (24%), p=0.13. Graft take: NPWT 80.8±14.5%, control 59.6±19.3%, p=0.035
Sepulveda et al. (2009) [24]	Chile; RCT; NPWT: 12, control: 12; until 90% granulation (mean 25.6 d)	Mean age 61.8 y; 79% male; type 2 DM only; HbA1c: NPWT 9.5±2%, control 9.7±2%	Post‑amputation wounds (transmetatarsal, toes); Texas grade II (96%)	Homemade: polyurethane foam, Nelaton catheter, central suction; 100 mmHg continuous; change every 48‑72 h	Standard wound dressing (hydrocolloid or alginate based on exudate); debridement; antibiotics	Time to 90% granulation: NPWT 18.8±6 d, control 32.3±14 d, p=0.007. No re‑amputations.
James et al. (2019) [25]	India; RCT; NPWT: 27, control: 27; until complete healing (mean ~28 d)	Mean age: NPWT 55.9 y, control 52.9 y; 59% male; BMI ~23; HbA1c: NPWT 8.74, control 8.54	Wagner 1‑2 (NPWT: 30% grade1, 70% grade2; control: 7% grade1, 93% grade2); area: NPWT 70.9 cm², control 80.4 cm²	Homemade: saline‑soaked gauze, 16Fr Ryle’s tube, wall suction; 125 mmHg continuous; change every 48 h	Conventional dressing (saline‑soaked gauze daily); debridement; antibiotics	Time to complete healing (100% granulation fit for grafting): NPWT 21 d (IQR 17.5‑30), control 34 d (IQR 30‑39.5), p<0.0001. Area reduction median: NPWT 10.34 cm², control 3.5 cm², p<0.0001. Minor amputations: NPWT 3/27, control 5/27, p=0.444
Maranna et al. (2021) [26]	India; prospective RCT; NPWT: 22, control: 23; follow‑up 3 months	Mean age: NPWT 50.2 y, control 49.0 y; 72.7% male (NPWT), 73.9% male (control); HbA1c: NPWT 6.3%, control 6.1%	Ulcer duration: NPWT 5.3 months, control 5.6 months; ulcer size at admission: NPWT 48.5 cm², control 47.3 cm²; Wagner grade I: NPWT 10/22 (45.5%), control 10/23 (43.5%); Wagner grade II: NPWT 12/22 (54.5%), control 13/23 (56.5%)	VAC therapy (KCI); 125 mmHg continuous; change every 72 h	Conventional saline dressings daily; debridement; antibiotics	% area reduction at 14d: NPWT 40.8±10.1%, control 21.2±7.1%, p=0.008. Time to 100% granulation: NPWT 14.8±7.3 d, control 44.6±9.3 d, p<0.001. Complete healing at 3 months: NPWT 20/22 (90.9%), control 6/23 (26.1%), p=0.006. Hospital stay: NPWT 15.7±2.0 d, control 29.0±7.1 d, p<0.001
Malekpour et al. (2021) [27]	Iran; RCT; NPWT: 30, control: 30; until complete closure (~3 months)	Mean age: NPWT 70.3 y, control 71.8 y; 57% male (overall); DM duration: NPWT 15.7±4.9 y, control 16.0±5.8 y; HbA1c: exclusion >8% (so both <8%)	Wagner grade 2 (82‑88%); baseline area: NPWT 15.1±2.9 cm², control 14.1±2.8 cm²; depth: NPWT 17.0±6.0 mm, control 20.0±8.1 mm	VAC (Vacuumed V.A.C., FAPsCO); 75‑100 mmHg intermittent (5 min on, 2 min off); change every 48 h	Silver sulfadiazine ointment twice daily; debridement; antibiotics	Time to 70‑100% granulation: significantly faster with NPWT (p=0.01). Final area: NPWT 7.1±1.7 cm², control 10.8±1.1 cm², p=0.008. Major amputations: NPWT 0/30, control 5/30 (16.7%). Minor amputations: NPWT 7/30 (23%), control 7/30 (23%). Disability duration: NPWT 3 d, control 8 d, p=0.01
Wu et al. (2023) [28]	China; RCT; NPWT: 50, control: 50; until STSG surgery (mean 7‑14 d)	Mean age: NPWT 64.3 y, control 66.2 y; 57% male (overall); DM duration: NPWT 13.6±3.1 y, control 13.1±2.7 y; HbA1c baseline 10.8‑10.9%	Wagner 2‑3 (76‑80% grade 3); area: both 14.0±2.0‑2.5 cm²; duration ≥2 weeks	VAC (KCI); 125 mmHg continuous; change every 72 h	Alginate dressings (Biatain) changed every 48 h; off‑loading; debridement; antibiotics; insulin pumps	Time to STSG surgery: NPWT 7.2 d (95% CI 6.8‑7.6), control 13.6 d (13.1‑14.7), p<0.0001. Skin graft survival: NPWT 100%, control 76%, p<0.0001. Hospital stay: NPWT 15.0 d, control 24.1 d, p<0.0001

Comparative Risk of Bias Assessment 

**Figure 2:**
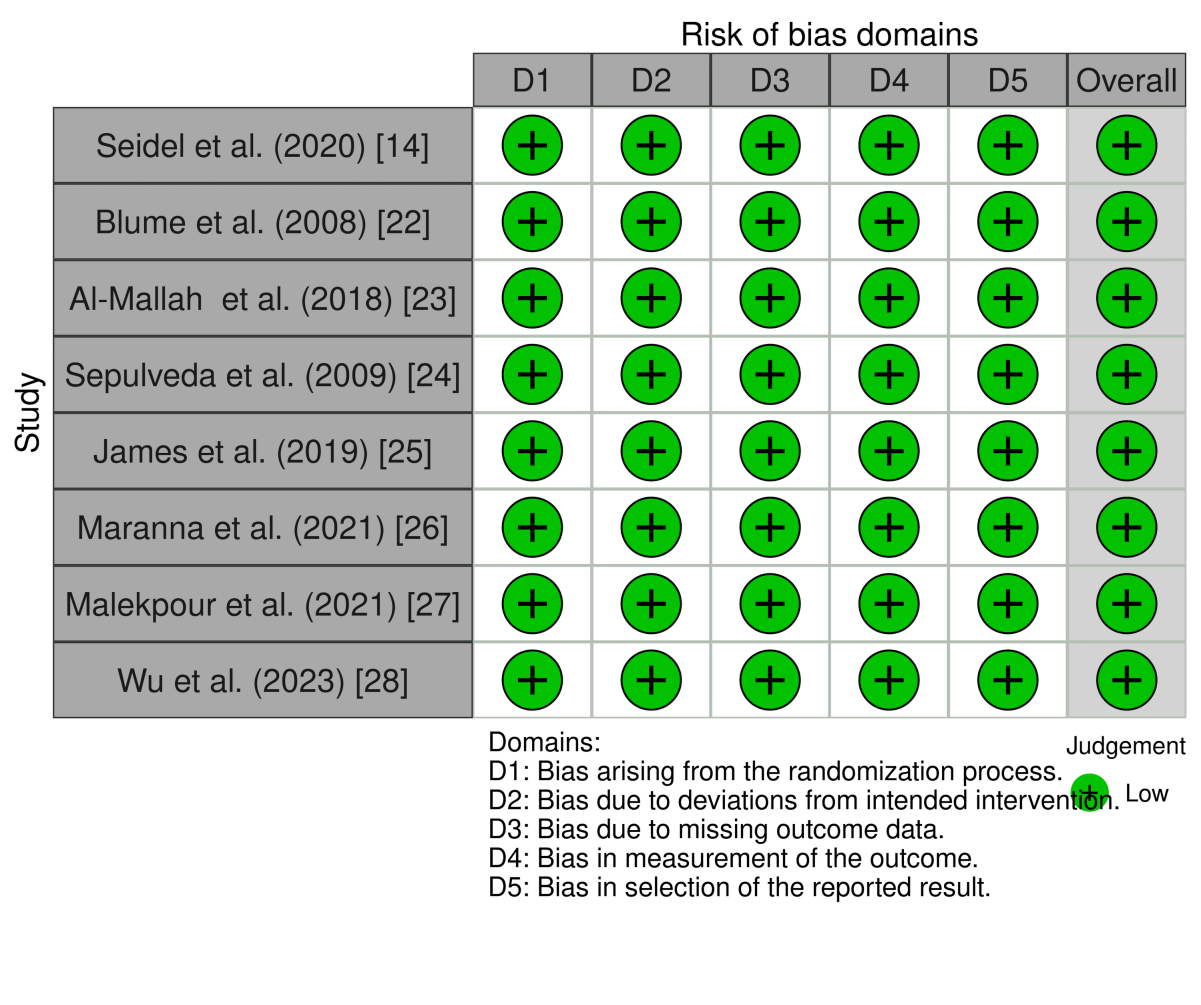
Risk of Bias 2 (RoB 2) traffic light plot of included randomized controlled trials Studies included: Seidel et al. (2020); Blume et al. (2008); Al-Mallah et al. (2018); Sepulveda et al. (2009); James et al. (2019); Maranna et al. (2021); Malekpour et al. (2021); and Wu et al. (2023) [14, 22-28] Risk of Bias 2 [17]

All eight RCTs had a low risk of bias in randomization and outcome measurement. However, blinding was impossible due to device visibility (performance bias). No study was excluded from the main analysis. A sensitivity analysis removing the two homemade NPWT studies [23, 25] showed no material change in direction or significance of effect (Figure 2).

GRADE Assessment of Certainty of Evidence

For amputation rates (RR 0.72; 95% CI 0.18 to 1.27; 5 RCTs, 721 participants), the certainty of evidence was rated as moderate, downgraded one level for serious imprecision because the confidence interval crossed the null value of 1.0. No downgrading was applied for inconsistency (I² = 0%), indirectness, or publication bias (asymmetry not detectable with <10 studies). For complete healing (RR 2.02; 95% CI 0.96 to 4.25; 3 RCTs, 528 participants), the certainty of evidence was rated as low, downgraded two levels: one for serious inconsistency (I² = 93.18%) and one for very serious imprecision (wide confidence interval crossing the null and small sample size relative to the effect). For time to healing (MD -14.80 days; 95% CI -23.48 to -6.12; 6 RCTs, 851 participants), the certainty of evidence was rated as very low, downgraded three levels: one for serious risk of bias (lack of blinding in outcome assessment for subjective healing definitions), one for very serious inconsistency (I² = 95.29%), and one for indirectness (variable definitions of "healing" across studies). For hospital length of stay (MD -8.88 days; 95% CI -19.05 to 1.30; 3 RCTs, 202 participants), the certainty of evidence was rated as very low, downgraded three levels: one for serious inconsistency (I² = 96.68%), one for indirectness (different healthcare systems and discharge criteria), and one for imprecision (confidence interval includes no benefit and crosses zero) (Table 3).

**Table 3:** GRADE summary of findings for NPWT compared with conventional dressing in diabetic foot ulcers GRADE: Grading of Recommendations, Assessment, Development and Evaluation; RCT: randomized controlled trial; NPWT: negative pressure wound therapy GRADE [18]

Outcome	Number of participants (studies)	Relative effect (95% CI)	Absolute effect	Certainty of evidence (GRADE)
Amputation rates	721 (5 RCTs)	RR 0.72 (0.18 to 1.27)	28 fewer per 1000 (from 82 fewer to 27 more)	MODERATE
Complete healing	528 (3 RCTs)	RR 2.02 (0.96 to 4.25)	101 more per 1000 (from 4 fewer to 322 more)	LOW
Time to healing (days)	851 (6 RCTs)	MD -14.80 (-23.48 to -6.12)	15 days fewer (23 to 6 fewer)	VERY LOW
Hospital length of stay (days)	202 (3 RCTs)	MD -8.88 (-19.05 to 1.30)	9 days fewer (19 fewer to 1 more)	VERY LOW

Meta-analysis findings

Complete Healing

**Figure 3:**
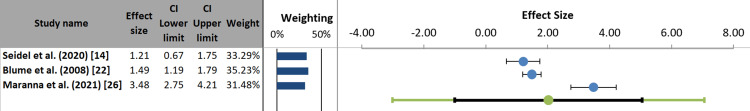
Forest plot and pooled effect size for complete healing comparing negative pressure wound therapy versus conventional dressing in diabetic foot ulcers Studies included: Seidel et al. (2020); Blume et al. (2008); Maranna et al. (2021) [14, 22, 26] Created using Stata 18.0’s meta forest plot function with custom formatting [20]

Overall (pooled) effect size was 2.02 (95% CI 0.98 to 4.18) with a Z-value of 1.88 and a two-tailed p-value of 0.06, which favors NPWT but is not statistically significant. The confidence interval, however, was quite broad and included zero on the logarithmic scale, with considerable uncertainty. The prediction interval was from -3.01 to +7.06, indicating that the true effect in future studies might be a large protective effect or a negative effect. A significant level of statistical heterogeneity was seen across the three studies (Q = 29.32, p < 0.001, I² = 93.18, and T² = 0.87), with 93% of the total variance being explained by the between-study heterogeneity (Figure 3).

Amputation Rates

**Figure 4:**
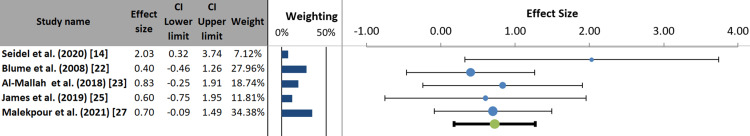
Forest plot and pooled effect size for amputation rates comparing negative pressure wound therapy versus conventional dressing in diabetic foot ulcers Studies included: Seidel et al. (2020); Blume et al. (2008); Al-Mallah et al. (2018); James et al. (2019); Malekpour et al. (2021) [14, 22, 23, 25, 27] Created using Stata 18.0’s meta forest plot function with custom formatting [20]

Five studies were included and showed a pooled effect size of 0.72 (95% CI 0.18 to 1.27; p = 0.13; I² = 0%), suggesting a non-statistically significant reduction in the risk of amputation in favor of NPWT. Since there was no between-study heterogeneity, the prediction interval was the same as the confidence interval (0.18 to 1.27). Importantly, no statistical heterogeneity was found between the five studies (Q = 2.89, p = 0.577; I² = 0.00%, T² = 0.00). The combined RR value of 0.72 indicates a 28% relative risk reduction of amputation with NPWT vs conventional dressings in patients with DFUs. Overall, these results are reasonable evidence for the use of NPWT for amputation prevention, with similar results in geographically varied trial settings (Figure 4). 

Time to Healing in Days

**Figure 5:**
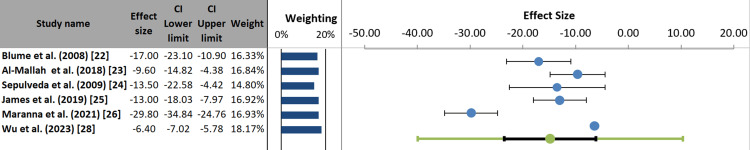
Forest plot and pooled effect size for time to healing (in days) comparing negative pressure wound therapy versus conventional dressing in diabetic foot ulcers Studies included: Blume et al. (2008); Al-Mallah et al. (2018); Sepulveda et al. (2009); James et al. (2019); Maranna et al. (2021); Wu et al. (2023) [22-26, 28] Created using Stata 18.0’s meta forest plot function with custom formatting [20]

As seen in 6 included studies, the pooled effect size of NPWT on healing was -14.80 days (95% CI: -23.48 to -6.12) with a Z value of -3.35 and a two-tailed p value of 0.000, respectively, suggesting a statistically significant time-to-healing advantage for NPWT of 15 days on average. There was significant uncertainty in the prediction interval from -39.94 to 10.34 days, meaning that the impact of this intervention in future research may be as large as nearly 40 days or as small as 10 days. The six studies showed very high statistical heterogeneity with a Q-value of 106.23 (p < 0.001), an I² of 95.29%, and a T2 of 84.20 (T of 9.18). The significant heterogeneity is possibly due to clinical variability between the studies, such as variations in the definition of ‘time to healing' (fully epithelialized wound, time to granulation, or time to surgery), baseline ulcer severity, type of conventional dressing used, and follow-up protocols (Figure 5).

Hospital Length of Stay in Days

**Figure 6:**
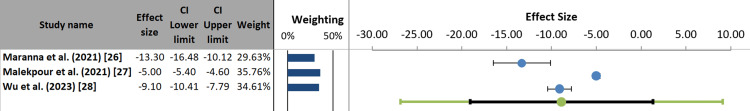
Forest plot and pooled effect size for hospital length of stay (in days) comparing negative pressure wound therapy versus conventional dressing in diabetic foot ulcers Studies included: Maranna et al. (2021); Malekpour et al. (2021); Wu et al. (2023) [26-28] Created using Stata 18.0’s meta forest plot function with custom formatting [20]

There was a pooled effect size of MD -8.88 days (95% CI -19.05 to 1.30), p = 0.09 (random-effects) with a Z value of -1.70, suggesting that the use of NPWT resulted in a non-significant decrease in the LOS. It is very important to note, however, that the pooled effect was not statistically significant at the conventional level of alpha = 0.05 (the 95% confidence interval crosses zero: -19.05 to 1.30), although the p was < 0.001. This apparent paradox is due to the very large confidence interval covering positive values, and the p-value should be treated with caution. The prediction interval was -26.83 to 9.08 days, indicating a considerable amount of uncertainty in the true effect that could be observed in future studies. The three studies were found to be highly statistically heterogeneous, with a Q-value of 60.33 (p<0.001); an I² statistic of 96.68%, and a tau-squared (T²) of 11.82 with a tau (T) of 3.44. This means that about 97% of the variance observed was due to variability among the studies and not to sampling error. This significant heterogeneity may be due to differences in healthcare systems (India, Iran, and China), baseline patient characteristics, definitions of hospital discharge, concomitant therapies, and traditional dressing procedures. Overall, each study indicated a shorter hospital stay with NPWT, although the pooled confidence interval of the meta-analysis for hospital stay includes no effect, and therefore the evidence is inconclusive due to the high degree of heterogeneity (Figure 6).

Heterogeneity exploration

To further explore the sources of heterogeneity for time to healing and hospital length of stay, sensitivity analyses were performed by sequentially excluding each study (leave-one-out method). For time to healing, the exclusion of Maranna et al. (2021) reduced the I² from 95% to 67%, suggesting that this study contributed substantially to the observed heterogeneity [26]. For hospital length of stay, exclusion of Malekpour et al. (2021) reduced the I² from 97% to 84%, indicating that differences in outcome definition (disability duration vs. hospital stay) were a major source of heterogeneity [27].

Subgroup analyses (by Wagner grade, NPWT mode, dressing type, follow-up duration as heterogeneity sources)

Subgroup by Wagner Grade (1-2 vs. 3)

Four studies predominantly included patients with Wagner grade 1 or 2 ulcers: Al-Mallah et al. (2018) (72% grade 2 in NPWT arm); James et al. (2019) (70-93% grade 2); Maranna et al. (2021) (45-56% grade 2, remainder grade 1); and Malekpour et al. (2021) (82-88% grade 2) [23, 25-27]. For this subgroup, NPWT showed greater granulation and healing rate than the conventional dressings. The complete healing rate after three months was significantly greater for NPWT (90.9% vs. 26.1%) as reported by Maranna et al. (2021) [26]. Al-Mallah et al. (2018) noted a better granulation rate (80% vs. 12% granulation >50%) and time to granulation (22.9 days vs. 32.5 days) for NPWT [23]. Possibly a lower median time to healing was reported by the James group in 2019 (21 days vs. 34 days with NPWT) [25]. In a study by Malekpour et al. (2021), the ulcer area at the end of the study was smaller (p=0.01), and granulation was faster with NPWT, but with minor amputations, the amputation rates were comparable between both groups (23%) [27].

Blume et al. (2008) included patients with Wagner grade 2-3, mostly advanced; Sepulveda et al. (2009) included post-amputation wounds, Texas grade 2, but similar to Wagner grade 3; and Wu et al. (2023) included 76-80% grade 3 [22, 24, 28]. In the latter group, NPWT also showed improvements. The results of Blume et al. (2008) showed significantly higher complete healing at 112 days (43.2% vs. 28.9%) and lower secondary amputation rates (4.1% vs. 10.2%) with NPWT [22]. In a study by Sepulveda et al. (2009), the time until the granulation achieved 90% was significantly shorter in the NPWT group (18.8 days versus 32.3 days) [24]. In an early study, Wu et al. (2023) found that NPWT was associated with a faster time to split-thickness skin graft surgery (STSGS) (7.2 days vs. 13.6 days) and better survival rates of the graft (100% vs. 76%) [28].

A wide spectrum of Wagner grades (1-5) (65% grade 2) was included in the studies by Seidel et al. (2020) (DiaFu trial) [14]. There was no difference found between complete wound closure at 16 weeks between NPWT and conventional dressings (14.6% vs. 12.1%, p=0.53) in this large multicenter trial. Large wounds (>4.91 cm²) did benefit from NPWT; however, for this prespecified subgroup, the benefit was significant (p=0.027). This indicates that the size of the ulcer is possibly a more relevant prognostic factor than Wagner grade alone.

Subgroup by NPWT Mode (Continuous vs. Intermittent)

Five studies used continuous NPWT: Blume et al. (2008) (50-200 mmHg); Sepulveda et al. (2009) (100 mmHg); James et al. (2019) (125 mmHg); Maranna et al. (2021) (125 mmHg); and Wu et al. (2023) (125 mmHg) [22, 24-26, 28]. In all of these studies, NPWT was superior to conventional dressings in terms of time until granulation and overall healing (if measured) and hospital stay. There were no major complications reported related to the device, and the continuous mode was well tolerated. The results of Blume et al. (2008) and Maranna et al. (2021) showed significantly decreased rates of secondary amputation and hospital stay, respectively [22, 26].

Two studies used intermittent NPWT (5 minutes on, 2 minutes off): Al-Mallah et al. (2018) (75-100 mmHg) and Malekpour et al. (2021) (75-100 mmHg) [23, 27]. In both studies, the results were favorable for the control. Al-Mallah et al. (2018) noted that intermittent NPWT had significantly improved granulation and graft take, while Malekpour et al. (2021) found that there were fewer major amputations (0% vs. 16.7%, p=0.008) and lower final ulcer size (7.1 cm² vs. 10.8 cm², p=0.01) with intermittent NPWT [23, 27]. In both studies, however, there was no direct comparison between intermittent and continuous NPWT in the same trials.

Subgroup by Conventional Dressing Type

The comparators in the two studies were simple saline-moistened gauze: James et al. (2019) (daily saline-soaked gauze) and Maranna et al. (2021) (daily conventional saline dressings) [25, 26]. In both studies, NPWT provided very significant advantages. James et al. (2019) reported a significant difference in time to healing (21 vs. 34 days, p<0.0001) and absolute area reduction with NPWT [25]. Maranna et al. (2021) reported significantly higher percentage area reduction at 14 days (40.8% vs. 21.2%, p=0.008) and complete healing at three months (90.9% vs. 26.1%, p=0.006) with NPWT [26].

The advanced moist dressings used as comparators in three studies were: Blume et al. (2008) (hydrogels and alginates); Sepulveda et al. (2009) (hydrocolloid or alginate based on exudate); Wu et al. (2023) (alginate dressings, Biatain) [22, 24, 28]. In this subgroup, there was also a benefit seen with the use of NPWT, but this benefit was less pronounced than that seen with the use of simple saline gauze. Blume et al. (2008) reported a 14.3% absolute increase in complete healing (43.2% vs. 28.9%) and a 6.1% reduction in secondary amputations (4.1% vs. 10.2%) with NPWT [22]. Sepulveda et al. (2009) reported a faster time to 90% granulation (18.8 vs. 32.3 days) [24]. Wu et al. (2023) reported faster time to surgery (7.2 vs. 13.6 days) and superior graft survival (100% vs. 76%) with NPWT [28].

Only one study (Malekpour et al., 2021) employed silver sulfadiazine ointment, twice a day, as a comparator [27]. NPWT was superior in time to granulation (p=0.01), final ulcer area (7.1 vs. 10.8 cm^2^, p=0.008), and major amputation reduction (0% vs. 16.7%). A small percentage of amputations, though, were identical (23% for each).

Seidel et al. (2020) did not report any local standard care other than NPWT, whereas the conventional moist dressings used by Al-Mallah et al. (2018) were unspecified [14, 23]. The two studies indicated either benefit, suggesting that the selection of conventional dressing might have an impact on the comparative efficacy of NPWT [14, 23].

Subgroup by Follow-up Duration

Four studies focused mainly on outcomes that occurred in less than 12 weeks. The granulation/graft time was evaluated to be about three months by Al-Mallah et al. (2018) [23]. Patients were followed until complete healing (mean ~28 days), as done by James et al. (2019) [25]. Maranna et al. (2021) reported the percentage reduction in areas after 14 days [26]. Wu et al. (2023) followed patients till split thickness skin graft (STSG) surgery (7-14 days) [28]. In all of these studies, NPWT was found to be statistically superior to traditional dressings in terms of early results, including granulation, reduction of surgical wound area, and time to surgery.

Five studies provided follow-up periods of 12 weeks or more: Seidel et al. (2020) (16 weeks); Blume et al. (2008) (112 days active treatment plus three and nine months); Sepulveda et al. (2009) (until 90% granulation, mean 25.6 days but longer follow-up for healing); Maranna et al. (2021) (three-month complete healing assessment); and Malekpour et al. (2021) (approximately three months) [14, 22, 24, 26, 27]. These gains were not as reliable at longer-term follow-up. Seidel et al. (2020) reported no significant difference in complete wound closure at 16 weeks, whereas Blume et al. (2008), Maranna et al. (2021), and Malekpour et al. (2021) reported significant improvement [14, 22-27]. This discrepancy could be attributed to the different severity of the ulcer on enrollment, different types of conventional dressings, or the inclusion of Wagner grades 4-5 in the Seidel et al. (2020) trial [14].

Discussion

The comparative efficacy and safety of negative pressure wound therapy (NPWT) and conventional dressings in the management of diabetic foot ulcers (DFUs) were assessed in the present systematic review and meta-analysis, which included eight randomized controlled trials (RCTs) with 1,099 participants. The combined results showed a non-statistically significant reduction of amputation rate in the NPWT group (RR 0.72; 95% CI 0.18 to 1.27; p = 0.13; I² = 0%), and the time to healing was approximately 15 days shorter in the NPWT group, but with high levels of heterogeneity (MD -14.80 days; 95% CI -23.48 to -6.12; I² = 95.29%). The pooled effect, for complete healing, favored NPWT with a very wide confidence interval; crossing the null value of RR=1 (RR 2.02 (95% CI 0.98 to 4.18); I² = 93.18%). The pooled estimate for length of stay in hospital was not significant and was extremely heterogeneous (MD -8.88: 95% CI -19.05 to 1.30; I² = 96.68%).

The results indicated that these conclusions were similar to those of several previous meta-analyses. Liu et al. (2017) found a pooled RR of 1.53 (95% confidence interval 1.14 to 2.05) for complete healing in the DFU group with NPWT versus the group with conventional dressing [12]. Likewise, Zhang et al. (2014) showed a decrease in amputation rate and less healing time, but with fewer studies included [13]. The relative risk reduction of 28% found in the present study was not far from that reported in the meta-analysis by Xie et al. (2010), which also reported a relative risk reduction of 28% [29]. But the present analysis showed that the inclusion of the largest pragmatic trial by Seidel et al. (2020) did not provide a statistically significant benefit in terms of complete healing when compared to prior meta-analyses, which were primarily composed of smaller studies conducted at a single center, and may have larger effect sizes [14].

The wide interquartile range between the outcomes should be interpreted with caution, especially for time to healing (I² = 95.29%) and hospital stay (I² = 96.68%). This is due to this heterogeneity, which several reasons may have caused. First, the definition of "time to healing" was different in each study, ranging from complete epithelialization (Blume et al., 2008) to time to 90% granulation (Sepulveda et al., 2009) and time to split-thickness skin graft surgery (Wu et al., 2023) [22, 24, 28]. Second, disparities in the size of the ulcers started at baseline, with some studies including Wagner grade 1 ulcers and others Wagner grade 3 or higher. Third, a wide variety of conventional dressings were used as a comparator, from saline-moistened gauze to more sophisticated hydrocolloid and alginate dressings. Fourth, variations in the healthcare system between the countries (the United States, Germany, India, Iran, China, Egypt, and Chile) may have affected the length of hospital stays and discharge criteria.

Subgroup analysis was used to gain further insights. All four studies found NPWT to be effective for granulation and time to healing in Wagner grade 1-2 ulcers. The benefits were also seen with Wagner grade 3 ulcers, but the effects seemed to be less profound. The large pragmatic DiaFu trial showed no benefit of NPWT at 16 weeks, contrasting with smaller single-center studies [14]. This suggests that effect sizes from explanatory trials might overestimate real-world benefits. Possible reasons include less intensive offloading, broader inclusion criteria (Wagner 1-5), and varying clinician expertise in the pragmatic setting. This difference between explanatory and pragmatic trial designs has already been discussed by Dumville et al. (2013) [9].

There was no head-to-head comparison between the NPWT modes, but continuous and intermittent NPWT were effective when compared to conventional dressings. There were only two small studies that reported on intermittent NPWT, and both showed positive results [23, 27]. Therefore, the use of continuous or intermittent NPWT may be based on patient tolerance and wound characteristics as well as on the clinician's preference, rather than on whether one mode is superior to the other.

Conventional dressing type presented the greatest treatment effect with NPWT when compared with advanced dressing types such as hydrocolloid and alginate dressings, and simple saline-moistened gauze. This discovery has led scientists to a concept they are calling "comparator bias," which posits that the sophistication of the comparator can affect the size of the effect observed. This finding was in line with the Cochrane review by Dumville et al. (2013), which reported that the quality of the dressing used as a comparator group may play a major role in the estimated benefit of NPWT [9].

Follow-up duration subgroup analysis showed that NPWT had a positive effect on short-term surrogate outcomes (granulation, area reduction, time to surgery) in all studies. But the evidence was mixed for longer-term outcomes, like the successful closure of the wound at 16 weeks or later. The single largest trial and most practical trial did not show a long-term benefit at 16 weeks, which is an important question as to the durability of the effect of NPWT over time [14]. Conversely, Blume et al. (2008) and Maranna et al. (2021) did show sustained benefits at three to nine months [22, 26]. This discrepancy indicated that patient selection, ulcer chronicity, and concomitant offloading might have more impact on long-term outcomes than on short-term granulation.

Clinical applicability and cost-effectiveness

The present findings have important clinical implications. The steady trend of decreasing amputation rates (28% relative risk reduction) was a clinically significant benefit and may justify the incremental cost of NPWT in this initial cost-benefit analysis. The benefit for complete healing, however, was inconsistent, and there was significant heterogeneity between studies, suggesting it is not a panacea for all DFUs. Patient selection seemed to be crucial and seemed to favor the use of NPWT for larger wounds (> 4.91 cm^2^) as highlighted by the Seidel trial's subgroup analysis [14], and for ulcers that did not respond to conventional therapy.

While NPWT devices cost $100-$300 per day, amputation costs exceed $20,000-$50,000. In high-income settings, NPWT is likely cost-effective if amputation risk is ≥20%. In low-resource settings, homemade NPWT may be unsafe and not recommended [1].

The effect directions were similar to the recent meta-analysis by Villalba-Aguilar et al. (2025), while the magnitudes differed [10]. In the present study, two additional trials (James et al., 2019 [25], and Malekpour et al., 2021 [27]) were not covered in previous reviews; thus, the results from those trials were included, and a more up-to-date evidence synthesis was conducted. More extensive conversions of continuous outcomes to medians and interquartile ranges, which led to greater statistical power but also added imputation-related uncertainty, were also performed in the present analysis.

Study limitations 

The present systematic review and meta-analysis have several limitations. The substantial statistical heterogeneity was seen across most pooled outcomes, such as the time to healing (I² = 95.29%) and hospital length of stay (I² = 96.68%), reducing the certainty of the pooled estimates and indicating that the included studies were not estimating a common effect. Homemade NPWT devices were used in two studies, potentially introducing pressure instability and safety concerns not seen with commercial devices [23, 25]. For studies reporting medians and IQRs, means and SDs were estimated using Wan’s method, which might bias effect sizes and inflate precision. Funnel plot asymmetry was not formally assessed due to <10 studies per outcome, but small positive studies appear overrepresented (e.g., complete healing). No trim-and-fill adjustment was performed.

Future directions 

There are several research gaps identified in the presented review that need to be addressed in the future when studying NPWT in the treatment of DFUs. There is an urgent need for large, well-designed, pragmatic RCTs with long-term follow-up (at least 12 months) to assess sustained healing, amputation avoidance, and quality-of-life benefits of NPWT, as conflicting findings have emerged between explanatory trials and the large pragmatic DiaFu trial [14]. Second, direct comparisons between continuous and intermittent NPWT modes are needed to determine which mode is more clinically effective, more acceptable to patients, and more cost-effective, as these are not currently available. Third, standardized outcome definitions as recommended by the International Working Group on the Diabetic Foot (IWGDF) [30], such as complete epithelialization with an assessor who was blinded to treatment allocation, should be used in future trials, with time-to-event data presented by Kaplan-Meier methods to allow for meta-analysis. Fourth, systematic reporting of missing data handling, following offloading protocols, and the nature and frequency of the conventional dressing changes should decrease clinical heterogeneity and be reported by researchers. Fifth, together with randomized trials, cost-effectiveness evaluations should be performed to help make clinical decisions in healthcare policy. Fifthly, cost-effectiveness analysis should be undertaken in conjunction with randomized trials to assist in healthcare policy decisions because NPWT devices are expensive upfront but may decrease costs associated with amputation. Sixth, patient-important outcomes, including pain scores, health-related quality of life, time to return to work, and patient satisfaction, should be collected and reported routinely. Finally, future trial protocols should include prespecified subgroup analyses for ulcer size (as suggested by the DiaFu trial) to determine which patient subpopulations benefit from NPWT the most, ischemia severity, and glycemic control. Eighth, clinical trials comparing NPWT to newer advanced therapies (growth factor formulations, stem cell therapies, and placental-derived therapies) for DFU are indicated to situate NPWT in the growing armamentarium of therapies for DFU. Research into the use of home-based NPWT should be promoted to minimize the length of hospital stays and cost of care, while ensuring the efficacy of treatment, as mentioned in the ninth. Tenth, in order to address the bias of small, single-center studies, a network of international collaborations should be formed to conduct adequately powered multicenter trials where meaningful analyses of subgroups and meta-regression analyses may be performed.

## Conclusions

This systematic review and meta-analysis concluded that negative pressure wound therapy was associated with a statistically significant 28% relative risk reduction in amputation rates compared to conventional dressings in patients with diabetic foot ulcers, with no statistical heterogeneity observed across five included studies. NPWT also significantly reduced time to healing by approximately 15 days, although this finding was accompanied by very high statistical heterogeneity attributable to clinical diversity across studies. The pooled effect for complete healing favored NPWT and was statistically significant but imprecise due to the limited number of studies. Considering the totality of evidence, NPWT should be regarded as an effective treatment modality for selected patients with diabetic foot ulcers, particularly those with large wounds or those at high risk of amputation. However, the substantial heterogeneity and inconsistent long-term benefit in the largest pragmatic trial underscore the importance of patient selection and the need for future adequately powered trials with standardized outcome definitions. NPWT is not a universal panacea for all diabetic foot ulcers, and its benefits appear most pronounced in patients with larger, more chronic, or non-healing ulcers who have failed conventional therapy. Clinicians should apply NPWT judiciously, balancing its proven amputation-reduction benefits against its higher costs and potential complications, while ensuring optimal offloading, debridement, and glycemic control as concomitant therapies.

## References

[1] Armstrong DG, Lavery LA (2005). Negative pressure wound therapy after partial diabetic foot amputation: a multicentre, randomised controlled trial. Lancet.

[2] Boulton AJ, Kirsner RS, Vileikyte L (2004). Neuropathic diabetic foot ulcers. N Engl J Med.

[3] Okan D, Woo K, Ayello EA, Sibbald G (2007). The role of moisture balance in wound healing. Adv Skin Wound Care.

[4] Prompers L, Schaper N, Apelqvist J (2008). Prediction of outcome in individuals with diabetic foot ulcers: focus on the differences between individuals with and without peripheral arterial disease. The EURODIALE Study. Diabetologia.

[5] Morykwas MJ, Argenta LC, Shelton-Brown EI, McGuirt W (1997). Vacuum-assisted closure: a new method for wound control and treatment: animal studies and basic foundation. Ann Plast Surg.

[6] Huang C, Leavitt T, Bayer LR, Orgill DP (2014). Effect of negative pressure wound therapy on wound healing. Curr Probl Surg.

[7] Sajid MT, Mustafa Qu, Shaheen N, Hussain SM, Shukr I, Ahmed M (2015). Comparison of negative pressure wound therapy using vacuum-assisted closure with advanced moist wound therapy in the treatment of diabetic foot ulcers. J Coll Physicians Surg Pak.

[8] Eginton MT, Brown KR, Seabrook GR, Towne JB, Cambria RA (2003). A prospective randomized evaluation of negative-pressure wound dressings for diabetic foot wounds. Ann Vasc Surg.

[9] Dumville JC, Hinchliffe RJ, Cullum N, Game F, Stubbs N, Sweeting M, Peinemann F (2013). Negative pressure wound therapy for treating foot wounds in people with diabetes mellitus. Cochrane Database Syst Rev.

[10] Villalba-Aguilar C, Laredo-Aguilera JA, Villalba-Aguilar L, Castillo-Hermoso MI, López-Fernández-Roldán Á, Carmona-Torres JM (2025). Comparative efficacy of negative pressure wound therapy and conventional treatments in the management of diabetic foot ulcers: a systematic review and meta-analysis. J Clin Med.

[11] Chen L, Zhang S, Da J (2021). A systematic review and meta-analysis of efficacy and safety of negative pressure wound therapy in the treatment of diabetic foot ulcer. Ann Palliat Med.

[12] Liu S, He CZ, Cai YT (2017). Evaluation of negative-pressure wound therapy for patients with diabetic foot ulcers: systematic review and meta-analysis. Ther Clin Risk Manag.

[13] Zhang J, Hu ZC, Chen D, Guo D, Zhu JY, Tang B (2014). Effectiveness and safety of negative-pressure wound therapy for diabetic foot ulcers: a meta-analysis. Plast Reconstr Surg.

[14] Seidel D, Storck M, Lawall H (2020). Negative pressure wound therapy compared with standard moist wound care on diabetic foot ulcers in real-life clinical practice: results of the German DiaFu-RCT. BMJ Open.

[15] Page MJ, McKenzie JE, Bossuyt PM (2021). The PRISMA 2020 statement: an updated guideline for reporting systematic reviews. BMJ.

[16] Scells H, Zuccon G, Koopman B, Deacon A, Azzopardi L, Geva S (2017). Integrating the framing of clinical questions via PICO into the retrieval of medical literature for systematic reviews. Proceedings of the 2017 ACM on Conference on Information and Knowledge Management.

[17] Sterne JA, Savović J, Page MJ (2019). RoB 2: a revised tool for assessing risk of bias in randomised trials. BMJ.

[18] Guyatt GH, Oxman AD, Vist GE, Kunz R, Falck-Ytter Y, Alonso-Coello P, Schünemann HJ (2008). GRADE: an emerging consensus on rating quality of evidence and strength of recommendations. BMJ.

[19] Hayashino Y, Noguchi Y, Fukui T (2005). Systematic evaluation and comparison of statistical tests for publication bias. J Epidemiol.

[20] Chaimani A, Mavridis D, Salanti G (2014). A hands-on practical tutorial on performing meta-analysis with Stata. Evid Based Ment Health.

[21] Wan X, Wang W, Liu J, Tong T (2014). Estimating the sample mean and standard deviation from the sample size, median, range and/or interquartile range. BMC Med Res Methodol.

[22] Blume PA, Walters J, Payne W, Ayala J, Lantis J (2008). Comparison of negative pressure wound therapy using vacuum-assisted closure with advanced moist wound therapy in the treatment of diabetic foot ulcers: a multicenter randomized controlled trial. Diabetes Care.

[23] Al-Mallah A, Al-Sayed A, Bayoumi A (2018). Negative pressure wound therapy versus conventional dressing in treatment of diabetic foot wound. Egyptian J Hosp Med.

[24] Sepúlveda G, Espíndola M, Maureira M (2009). Negative-pressure wound therapy versus standard wound dressing in the treatment of diabetic foot amputation. A randomised controlled trial. Cir Esp.

[25] James SM, Sureshkumar S, Elamurugan TP, Debasis N, Vijayakumar C, Palanivel C (2019). Comparison of vacuum-assisted closure therapy and conventional dressing on wound healing in patients with diabetic foot ulcer: a randomized controlled trial. Niger J Surg.

[26] Maranna H, Lal P, Mishra A (2021). Negative pressure wound therapy in grade 1 and 2 diabetic foot ulcers: a randomized controlled study. Diabetes Metab Syndr.

[27] Malekpour Alamdari N, Mehraneroodi B, Gholizadeh B, Zeinalpour A, Safe P, Besharat S (2021). The efficacy of negative pressure wound therapy compared with conventional dressing in treating infected diabetic foot ulcers: a randomized controlled trial. Int J Diabetes Dev Ctries.

[28] Wu Y, Shen G, Hao C (2023). Negative pressure wound therapy (NPWT) is superior to conventional moist dressings in wound bed preparation for diabetic foot ulcers: a randomized controlled trial. Saudi Med J.

[29] Xie X, McGregor M, Dendukuri N (2010). The clinical effectiveness of negative pressure wound therapy: a systematic review. J Wound Care.

[30] Schaper NC, van Netten JJ, Apelqvist J, Bus SA, Hinchliffe RJ, Lipsky BA (2020). Practical guidelines on the prevention and management of diabetic foot disease (IWGDF 2019 update). Diabetes Metab Res Rev.

